# A phase II study of capecitabine and oxaliplatin combination chemotherapy in patients with inoperable adenocarcinoma of the gall bladder or biliary tract

**DOI:** 10.1186/s13104-015-1778-4

**Published:** 2016-03-12

**Authors:** J. S. Graham, K. Boyd, F. Y. Coxon, L. R. Wall, M. M. Eatock, T. S. Maughan, M. Highley, E. Soulis, S. Harden, P. Bützberger-Zimmerli, T. R. J. Evans

**Affiliations:** Beatson West of Scotland Cancer Centre, 1053 Great Western Road, Glasgow, G12 OYN UK; Northern Centre for Cancer Care, Freeman Hospital, Newcastle upon Tyne, NE7 7DN UK; Edinburgh Cancer Centre, Western General Hospital, Edinburgh, EH4 2XU UK; Belfast Cancer Centre, Belfast City Hospital, Belfast, BT9 7AB UK; Velindre Hospital, Whitchurch, Cardiff, CF14 2TL UK; Ninewells Hospital, Dundee, DD1 9SY UK; Institute of Cancer Sciences, University of Glasgow, Glasgow, G61 1BD UK

**Keywords:** Gall bladder, Biliary tract, Capecitabine, Oxaliplatin

## Abstract

**Background:**

Advanced biliary tract carcinomas are associated with a poor prognosis, and palliative chemotherapy has only modest benefit. This multi-centre phase II study was conducted to determine the efficacy of capecitabine in combination with oxaliplatin in patients with inoperable gall bladder or biliary tract cancer.

**Methods:**

This was a Phase II, non-randomised, two-stage Simon design, multi-centre study. Ethics approval was sought and obtained by the North West MREC, and then locally by the West Glasgow Hospitals Research Ethics Committee. Eligible patients with inoperable locally advanced or metastatic adenocarcinoma of the gall bladder or biliary tract and with adequate performance status, haematologic, renal, and hepatic function were treated with capecitabine (1000 mg/m^2^ po, twice daily, days 1–14) and oxaliplatin (130 mg/m^2^ i.v., day 1) every 3 weeks for up to six cycles. The primary objective of the study was to determine the objective tumour response rates (complete and partial). The secondary objectives included assessment of toxicity, progression-free survival, and overall survival.

**Results:**

Forty-three patients were recruited between July 2003 and December 2005. The regimen was well tolerated with no grade 3/4 neutropenia or thrombocytopenia. Grade 3/4 sensory neuropathy was observed in six patients. Two-thirds of patients received their chemotherapy without any dose delays. Overall response rate was 23.8 % (95 % CI 12.05–39.5 %). Stable disease was observed in a further 13 patients (31 %) and progressive disease observed in 12 (28.6 %) of patients. The median progression-free survival was 4.6 months (95 % CI 2.8–6.4 months; Fig. 1) and the median overall survival 7.9 months (95 % CI 5.3–10.4 months; Fig. 2).

**Fig. 1:**
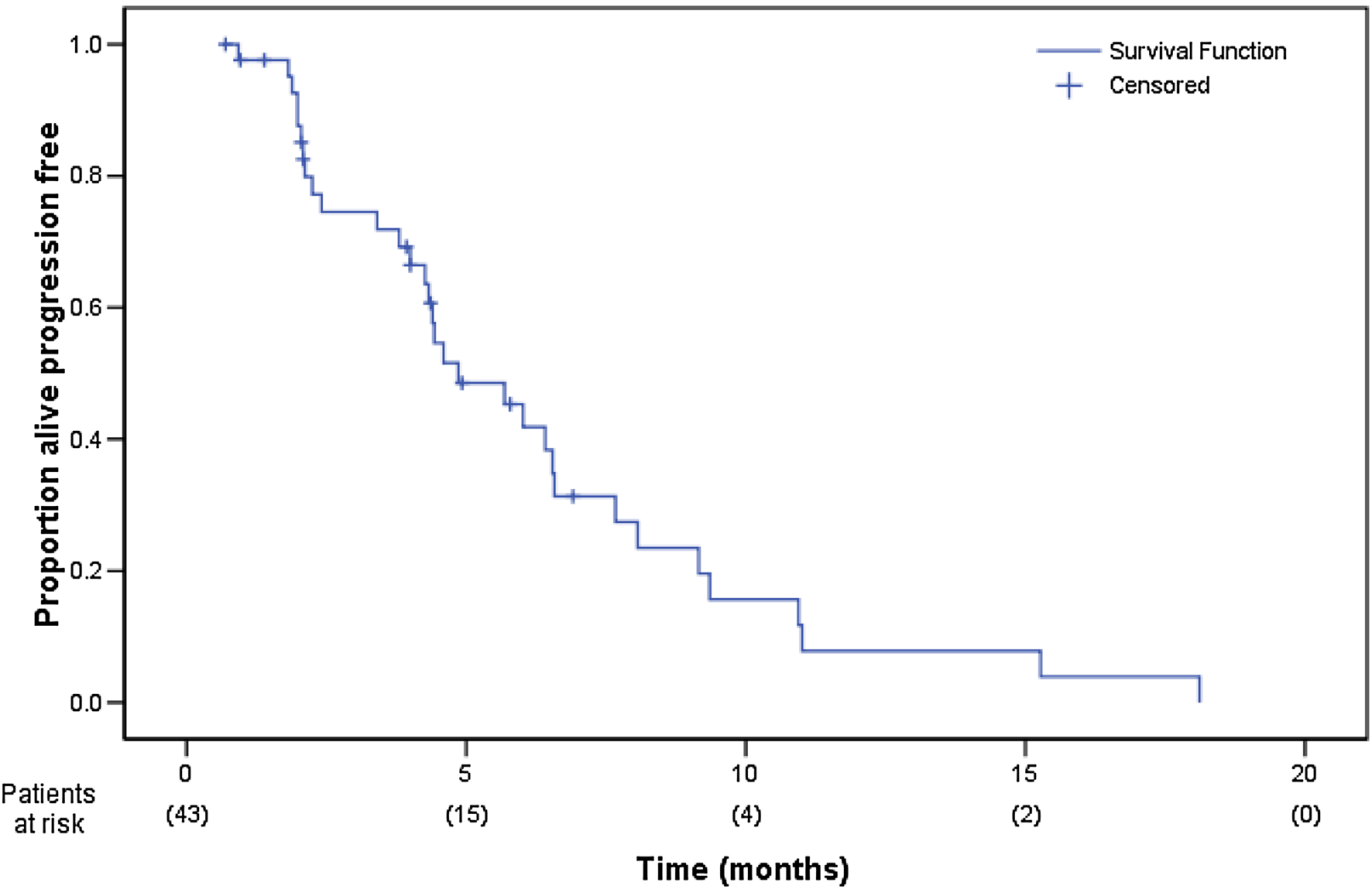
Progression-free survival

**Fig. 2:**
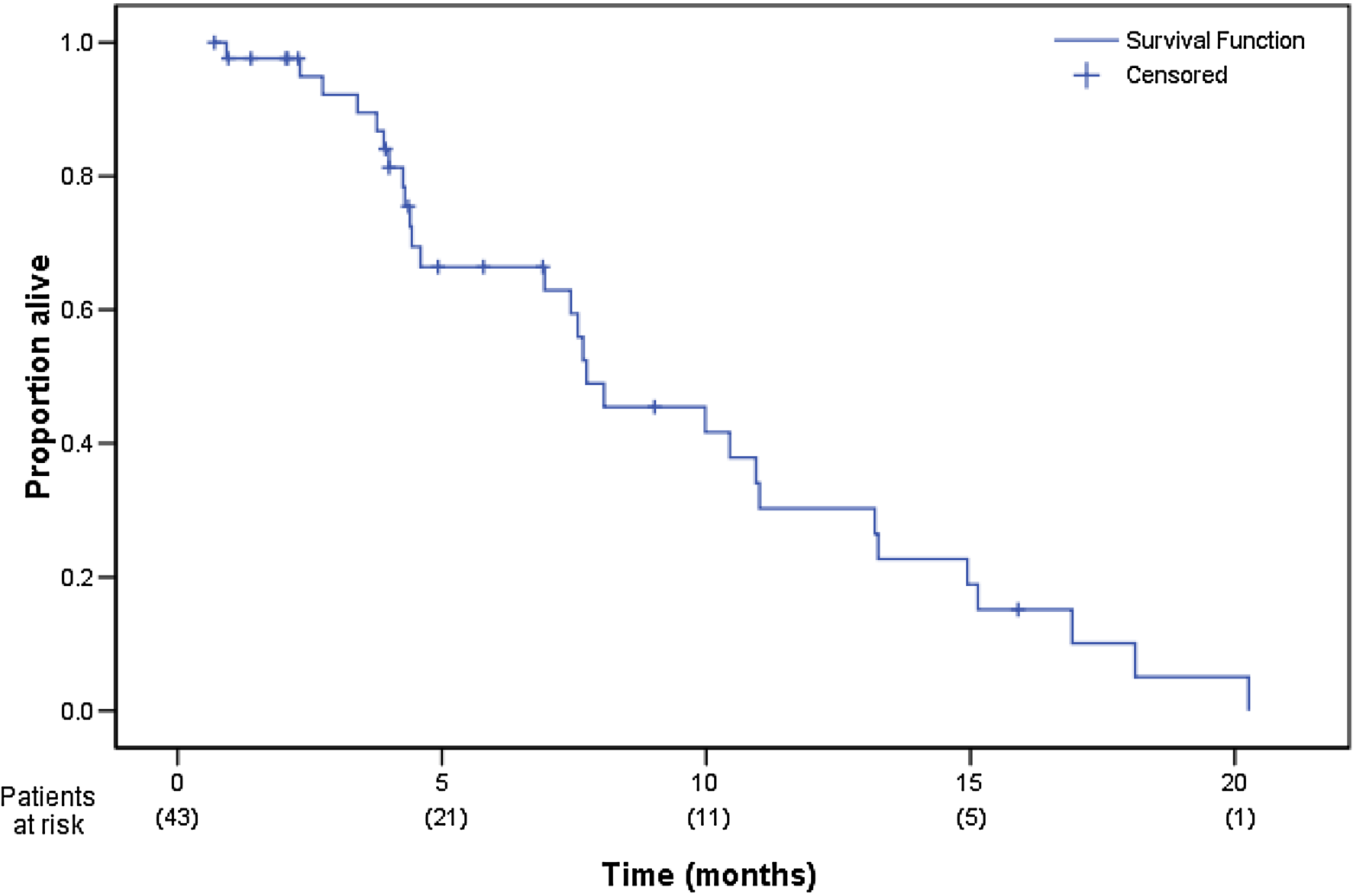
Overall survival

**Conclusion:**

Capecitabine combined with oxaliplatin has a lower disease control and shorter overall survival than the combination of cisplatin with gemcitabine which has subsequently become the standard of care in this disease. However, capecitabine in combination with oxaliplatin does have modest activity in this disease, and can be considered as an alternative treatment option for patients in whom cisplatin and/or gemcitabine are contra-indicated.

## Background

Carcinomas of the gall bladder and biliary system are relatively rare malignancies accounting for approximately 4–5 % of all gastrointestinal cancers in Europe and the USA. Patients with these tumours often present with advanced disease, and so curative surgical options are limited and survival rates poor. Numerous cytotoxic agents have been evaluated both as single agent therapy and combination chemotherapy regimens (reviewed in [1]), but response rates in excess of 30 % have been difficult to achieve [1]. Nevertheless, chemotherapy can result in a significantly improved survival and an improved quality of life compared with best supportive care [2].

One of the most extensively studied cytotoxic agents in biliary tract cancer is 5-fluorouracil (5-FU), either alone or in combination. Response rates of 10–24 % have been reported for 5-FU as single agent [3–6], and of 10–40 % when used in combination [1], including 40 % with the ECF regimen, although most of these studies have been in small numbers of patients. In addition, there were no significant differences between single agent 5-FU and the FAM combination regimen in terms of median time to disease progression or median survival in randomised trials [7]. At the time of conducting this study, no standard therapy existed for advanced gall bladder or biliary tract cancer, with gemcitabine monotherapy or the combination of cisplatin with 5-FU among the most widely used regimens outside of clinical trials. However, the response rate for cisplatin in combination with 5-FU is only approximately 30–35 %. Consequently, further clinical trials of novel chemotherapy agents are warranted in gall bladder and biliary tract cancer.

Capecitabine is an oral fluoropyrimidine pro-drug with preferential conversion to 5-FU in tumour tissue compared to normal by exploiting the increased intra-tumoural expression of thymidine phosphorylase [8], and which is now extensively used in the treatment of colon [9–15], breast [16, 17] and gastric cancers [18]. Oxaliplatin is a third-generation cisplatin analogue, with activity and toxicity profiles that differ from those of other platinum derivatives, including carboplatin and cisplatin [19] and with clinical activity, either alone or in combination with 5-FU, in advanced colorectal cancer [9, 11–14, 20].

When the combination of capecitabine with oxaliplatin was evaluated in the phase I study, [12] a patient with gall bladder carcinoma who had progressed through treatment with a combination of 5-FU and leucovorin, had a partial response when treated with XELOX. This, taken together with the activity of single-agent 5-FU and of 5-FU in combination with cisplatin in biliary tract cancer, led us to explore the activity of a combination of capecitabine and oxaliplatin as a first-line therapy regimen within a phase II study in biliary tract cancers..

## Patients and methods

### Study design

This was a Phase II, non-randomised, two-stage Simon design, multi-centre study of capecitabine and oxaliplatin combination chemotherapy in patients with inoperable locally advanced or metastatic adenocarcinoma of the gall bladder or biliary tract. The primary objective of the study was to determine the objective tumour response rates (complete and partial), and secondary objectives included assessment of toxicity, progression-free survival, and overall survival. The study was approved by the North West MREC (Multi-Centre Research Ethics Committee) and then by the local research committees and NHS R&D departments of each participating institution. All patients gave written, informed, consent prior to any study related procedure.

### Eligibility criteria

Eligible patients were those with histologically or cytologically confirmed adenocarcinoma of the gall bladder or biliary tract, with inoperable disease as determined by radiological assessment, laparotomy or laparoscopy. All patients had measurable disease, as defined by the response evaluation criteria in solid tumours criteria [RECIST] [21], with ECOG performance status ≤2, were at least 18 years of age, and had adequate renal (serum creatinine ≤1.5× institution’s upper limit of normal [ULN]), hepatic (bilirubin ≤2.5× ULN; transaminases ≤5× ULN), and haematological (absolute neutrophil count [ANC] ≥ 1500/mm^3^; platelet counts ≥ 100,000/mm^3^; haemoglobin ≥ 10 g/dl) function.

Patients were excluded if they had received any prior chemotherapy for metastatic disease, if they had received any radiotherapy, hormonal or immunotherapy within 4 weeks prior to study entry, or if they were unlikely to tolerate treatment. Other exclusion criteria included patients who were pregnant or of child-bearing potential and unwilling to use an acceptable method of birth control, peripheral sensory neuropathy of >grade 1 of any aetiology, known hypersensitivity to fluoropyrimidines or oxaliplatin, and any evidence of uncontrolled cardiac disease or any other serious medical or psychiatric disorder that would be a contra-indication for prescribing this chemotherapy regimen. Patients were also excluded if they will unable to reliably tolerate and comply with oral medication or if they had a lack of physical integrity of the GI tract leading to a malabsorption syndrome or intestinal obstruction that would impair administration or absorption of oral therapy.

### Treatment administration

Capecitabine was administered in an intermittent schedule (14 days’ treatment; 7 days’ rest period) at a dose of 1000 mg/m^2^ twice daily orally in a 21-day treatment cycle. The two daily doses of capecitabine were administered 12 ± 2 h apart, within 30 min after a meal with approximately 200 ml of water. The total daily dose was “rounded-up” and given in equally divided twice daily doses. Patients were provided with a study diary card to record drug administration.

Oxaliplatin was administered on day 1 at a dose of 130 mg/m^2^ as a 2-hour intravenous infusion, after the morning dose of capecitabine, in a 21-day treatment schedule. If patients developed acute, laryngo-pharyngeal dysesthesiae, the subsequent doses of oxaliplatin were administered as a 6-h infusion.

Treatment was repeated every 21 days for a maximum of six courses, but was discontinued prior to this if there was evidence of disease progression, intolerable toxicity despite dose modification, patient refusal, or investigator decision to discontinue study therapy. Patients received prophylactic anti-emetic medication prior to the oxaliplatin infusion with intravenous dexamethasone and granisetron, followed by oral dexamethasone and domperidone for 3 and 5 days respectively.

### Dose delays and dose modifications

Administration of subsequent courses of capecitabine and oxaliplatin was delayed for one week if non-haematological toxicity had not resolved to baseline levels or to ≤grade 1, or if the neutrophil count was <1.0 × 10^9^/l or if the platelets were <75 × 10^9^/l. Chemotherapy could be delayed for a maximum of 2 weeks to allow recovery from toxicity. If toxicity did not resolve after a delay of 2 weeks, then treatment was discontinued. Capecitabine was interrupted in patients who developed ≥grade 2 diarrhoea, stomatitis, or hand-foot syndrome, and doses of capecitabine omitted for toxicity were not replaced or restored. When toxicities had resolved to grade ≤1, capecitabine was recommenced with an appropriate dose modification as follows: a reduction of the daily dose by 25 % for first occurrence of grade 3 toxicity or second occurrence of grade 2 toxicity; a reduction of the daily dose by 50 % for grade 4 toxicity (first occurrence), grade 3 toxicity (second occurrence), or grade 2 toxicity (third occurrence). Capecitabine was discontinued for grade 4 toxicity (second occurrence), grade 3 toxicity (third occurrence), and grade 2 toxicity (fourth occurrence).

### Supportive care

Palliative and supportive care was permitted during the study, but patients who required radiotherapy during the study were considered to have disease progression and were withdrawn from the study. Patients were not permitted to receive any other anti-cancer therapy during the study (including hormonal agents and immunotherapy). The use of prophylactic granulocyte colony stimulating factor (G-CSF) was not permitted during the first two cycles of drug administration.

### Patient assessments

Patient assessments included clinical evaluation, toxicity assessments (NCI CTC version 2.0) and laboratory assessments every 3 weeks. Disease status was evaluated prior to starting chemotherapy and after three cycles of chemotherapy. Patients with stable disease or who were responding could receive a maximum of six cycles of chemotherapy, following which disease status was re-assessed within 4 weeks of the last cycle of chemotherapy. Disease assessment was by chest X-ray and CT scan of the abdomen, and other radiological assessments were performed as appropriate. Response was determined using RECIST [21]. Overall survival (all causes) was determined from the start of chemotherapy to the time of death and progression-free survival measured from the start of chemotherapy until subsequent disease progression.

### Statistical analyses

As responses have been observed with 5-FU, with capecitabine and with the combination of capecitabine with oxaliplatin within the phase 1 study, a Gehan design for this phase II study was not appropriate. Therefore using a two-stage Simon design, it was calculated that recruitment of a total of 43 patients would give 80 % power at the 5 % significance level of detecting a response rate of ≥40 %, at which point it would be appropriate to consider further studies with this regimen, and a response rate of ≤20 %, below which this regimen would not be pursued in subsequent studies. Thirteen patients would be recruited in the first stage; if three or fewer responses were observed during this stage then recruitment would be halted and no further investigation of the combination warranted. If 13 or more responses were observed by the end of the study the combination would merit further development.

## Results

Forty-three patients were recruited from six centres between July 2003 and December 2005. One patient was subsequently excluded from the analysis as they had a CVA and not received any treatment within the trial. Patient and tumour characteristics are shown in Table 1.

**Table 1:** Patient characteristics

Age, median (years)	60.5 (range 37–81)
Gender	
Male	24 (57.1)
Female	18 (42.9)
ECOG performance status	
0	9 (21.4)
1	27 (64.3)
2	6 (14.3)
Primary tumour	
Gallbladder	18
Bile ducts	22
Ampulla of vater	2
Metastatic disease at presentation	
Yes	39
No	3

A total of 158 courses of chemotherapy were administered in 42 patients (median = 3). Eleven patients (26.2 %) completed all six planned courses of chemotherapy. All analyses were performed on an intention to treat basis.

### Toxicity assessments

Haematological toxicity was minimal with no grade 3/4 neutropenia or thrombocytopenia, and only one occurrence of grade 3/4 anaemia. Grade 3/4 biochemical abnormalities (worst grade per patient, all cycles) included elevated alkaline phosphatase (10), bilirubin (3), glucose (3), and ALT, urea, and low albumin (all 1 each). Sensory neuropathy was observed in 37 patients, and was grade 3/4 in 6 patients. Other grade 3/4 toxicities included fatigue (6 patients), vomiting (5), nausea (4), diarrhoea (6), abdominal cramps (2), and anorexia (1) (maximum grade per patient).

### Dose delays and modifications

Twenty-five patients (59.5 %) received chemotherapy without any dose delays. A total of 25 cycles of chemotherapy were delayed for thrombocytopenia (3 cycles), neutropenia (1), and deranged liver function (1 cycle). Other reasons for dose delays (21 cycles) included laryngeal dysaethesia, diarrhoea, hypokalaemia, chest infection, spinal cord compression, DVT, a requirement for paracentesis, abdominal pain, biliary sepsis, pulmonary embolism and elective change of biliary stent.

Dose modification of capecitabine was required for toxicity in 21 patients and of oxaliplatin in 6 patients. The duration of infusion of oxaliplatin had to be prolonged in 5 patients for pharyngo- laryngeal dysaesthesia.

### Efficacy

With the exception of the patient who suffered a cerebrovascular event (CVA) and did not start treatment, all 42 patients were included in the efficacy analyses (intention–to–treat). There was one complete response (2.4 %) and 9 patients (21.4 %) had a partial response for an overall response rate of 23.8 % (95 % CI 12.05–39.5 %). Stable disease was observed in a further 13 (31 %) of patients, giving a disease control rate of 54.8 % (CR, PR and SD), with progressive disease observed in 12 (28.6 %) of patients (Table 2). Seven patients (16.7 %) did not have formal repeat assessments of disease status to determine response. Of these, five patients were withdrawn early due to treatment-related toxicities, one patient withdrew consent following the first cycle of treatment, and 1 patient died prior to cycle 2.

**Table 2:** Response assessment

Assessment of overall response in target and non-target lesions	Number of patients	%
CR	1	2.4
PR	9	21.4
SD	13	31.0
PD	12	28.6
Unevaluable, target lesions not assessed	7	16.7
Total	42	100

The median progression-free survival is 4.6 months (95 % CI 2.8–6.4 month; Fig. 1) and the median overall survival is 7.9 months (95 % CI 5.3–10.4 months; Fig. 2).

## Discussion

The aim of this study was to determine the objective response rate of a combination of capecitabine and oxaliplatin in patients with advanced gall bladder or biliary tract cancer. The hypothesis was that a regimen with an objective response rate of >40 % would warrant further investigation, while a regimen with a response rate of <20 % would not be of further interest. Six patients discontinued chemotherapy with this regimen prior to the first planned disease re-assessment due to toxicity, which is higher than the patient withdrawal rate with this regimen in other tumour types, although the grade 3/4 toxicities were comparable to previous reports of this regimen in other tumour types. In total, 10 patients did not undergo repeat disease assessments for response. Ten of 33 patients assessable for response had an objective tumour response for an overall response rate of 30.3 %. However, the objective response rate, based on an intention to treat, in this study was 23 %, and so we do not recommend further study of this regimen as a first-line therapy regimen patients with advanced gall bladder or biliary tract cancer.

A similar response rate (21 %; 95 % CI 9–38 %) was observed in another study in which two 3-weekly cycles of capecitabine (1000 mg/m^2^ bid, days 1–14) plus oxaliplatin (130 mg/m^2^ i.v., on day1) were followed by XELOX-RT (radiotherapy [50.4 Gy] combined with capecitabine [750–675 mg/m^2^ bid] every radiotherapy day and oxaliplatin [40–30 mg/m^2^] once weekly [22]). The maximum tolerated doses of oxaliplatin and capecitabine when combined with radiotherapy were 30 and 675 mg/m^2^, respectively. Five patients became operable following this chemo-radiotherapy regimen, with three R0 resections and this corresponded to a two-year survival of 28 %, and with an estimated local tumour control rate at 2 years of 72 %. Objective response rates of between 18 and 41 % have been reported with most other oxaliplatin–containing regimens [23–27]. Similarly, response rates of 6.6–40.6 % have been seen with a number of other combination chemotherapy regimens in this disease (Table 3).

**Table 3:** Chemotherapy studies in advanced biliary tract cancer

Author (year)	Drug	Number of patients	Response rate (%)
Taal (1993) [32]	MMC	30	10
Patt (1996) [33]	5FU	32	34
	Interferon alfa-2b		
Patt (2001) [34]	Cisplatin Interferon alpha-2b	41	21
	Doxorubicin		
	5 FU		
Penz (2001) [35]	Gemcitabine	32	22
Murad (2003) [36]	Gemcitabine	26	31
	5FU		
Kornek (2001) [37]	MMC/Capecitabine vs	51	31
	MMC/gemcitabine		20
Ueno (2004) [38]	S1	19	21
Alberts (2005) [39]	Gem	42	9.5
	5FU		
	Leucovorin		
Furuse (2006) [40]	Uracil-tegafur	24	12.5
	Doxorubicin		
Kim (2006) [41]	Gemcitabine	29	34.5
	Cisplatin		
Okusaka (2006) [42]	Gemcitabine	40	17.5
Park (2006) [43]	Gemcitabine	27	33.3
	Cisplatin		
Hong (2007) [44]	Capecitabine	32	40.6
	Cisplatin		
Manzione (2007) [23]	Gemcitabine	34	41
	Oxaliplatin		
Furuse (2008) [45]	S1	40	35
Kim (2008) [46]	S1	51	30
	Cisplatin		
Oh (2008) [27]	S1	15	6.7
	Oxaliplatin		
Furuse (2009) [47]	Uracil-tegafur	61	6.6
	Doxorubicin		
Kim (2009) [25]	Gemcitabine	40	15
	Oxaliplatin		
Sasaki (2009) [48]	S1	28	34.3
	Gemcitabine		
Gruenberger (2010) [30]	Cetuximab	30	63
	Gemcitabine		
	Oxaliplatin		
Gunnlaugsson (2010) [22]	Capecitabine	39	21
	Oxaliplatin		
Kanai (2010) [49]	S1	25	30.4
	Gemcitabine		
Karachaliou (2010) [24]	Irinotecan	28	17.9
	Oxaliplatin		
Chung (2011) [50]	Irinotecan	39	20.5
	Gemcitabine		

A further study [28] without radiotherapy was very similar to ours and looked at oxaliplatin (130 mg/m^2^) and capecitabine (1000 mg/m^2^ bid, days 1–14) 3 weekly as firstline treatment in advanced biliary carcinoma. 65 patients were stratified prospectively into two groups based on location of the primary (gallbladder or extra hepatic cholangiocarcinoma (GBC/ECC) versus intrahepatic mass-forming type cholangiocarcinoma (ICC)). The response rates overall were significantly higher in the GBC/ECC (27 % OR, 49 % SD) than ICC group (no objective response, 33 % SD). Median survival was 12.8 months (CI 95 % 10–16.8 months) for for GBC or ECC compared with 5.2 months in the ICC (CI 95 % 12.7–20.5 months). The morphologically distinct presentations appear to differ in their presentations and response to cytotoxic drugs [29]. Our study replicates similar response rates but our survival times were shorter (between those of ICC and GBC/ECC groups) which may reflect many reasons, such as overall health of the included patients; differences in such characteristics will be more apparent given the small numbers.

A GERCOR study [30] has looked at the combination of gemcitabine (1000 mg/m^2^ day 1) and oxaliplatin (100 mg/m^2^ day 2) fortnightly as firstline treatment in biliary tract carcinoma and observed (again with small numbers—56 total) similar response rates to ours but also that the combination was well tolerated in frailer patients (it included patients of performance status (PS) >2 in one of the two groups). Group A (PS 0–2, bilirubin <2.5× ULN) showed OR in 36 % (95 % CI 18.7–52.3 %), stable disease 26 %, progressive disease 39 %. Median PFS was 5.7 months and OS was 15.7 months. Group B (PS >2 and/or bilirubin >2.5× ULN) showed OR 22 % (95 % CI 6.5–37.4 %), stable disease 30 %, progressive disease 48 %. PFS was 3.9 months and OS 7.6 months. A two weekly regimen would mean slightly more hospital visits when compared with oxaliplatin and capecitabine but again may provide an alternative regimen to the current gold standard.

Following completion of our study, the combination of cisplatin (25 mg/m^2^) followed by gemcitabine (1000 mg/m^2^ days 1 and 8) every 3 weeks for eight cycles is now established as the standard of care in this disease following a phase III randomised trial in comparison with gemcitabine monotherapy. This regimen results in a disease control rate of 81.4 %, median progression-free survival of 8.0 months, and a median overall survival of 11.7 months [31]. The results of our study suggest that the combination of capecitabine with oxaliplatin has lower disease control and shorter overall survival than cisplatin in combination with gemcitabine. However, the combination of capecitabine and oxaliplatin remains a regimen with activity in this disease, and can be considered as an alternative treatment option for patients in whom cisplatin and/or gemcitabine are contra-indicated.
